# Effects of controlled diesel exhaust exposure on apoptosis and proliferation markers in bronchial epithelium – an *in vivo* bronchoscopy study on asthmatics, rhinitics and healthy subjects

**DOI:** 10.1186/s12890-015-0096-x

**Published:** 2015-08-25

**Authors:** Annelie F Behndig, Karthika Shanmuganathan, Laura Whitmarsh, Nikolai Stenfors, Joanna L Brown, Anthony J Frew, Frank J Kelly, Ian S Mudway, Thomas Sandström, Susan J Wilson

**Affiliations:** Department of Public Health and Clinical Medicine, Division of Medicine/Respiratory Medicine, Umeå University, Umeå, Sweden; Histochemistry Research Unit, Clinical and Experimental Sciences, Faculty of Medicine, University of Southampton, Southampton, UK; MRC – PHE Centre for Environment and Health, Franklin-Wilkins Building, King’s College London, London, UK; Histochemistry Research Unit, Sir Henry Wellcome Laboratories, Mailpoint 894, Level B, South Block, Southampton General Hospital, Tremona Road, Southampton, UK

## Abstract

**Background:**

Epidemiological evidence demonstrates that exposure to traffic-derived pollution worsens respiratory symptoms in asthmatics, but controlled human exposure studies have failed to provide a mechanism for this effect. Here we investigated whether diesel exhaust (DE) would induce apoptosis or proliferation in the bronchial epithelium *in vivo* and thus contribute to respiratory symptoms.

**Methods:**

Moderate (*n* = 16) and mild (*n* = 16) asthmatics, atopic non-asthmatic controls (rhinitics) (*n* = 13) and healthy controls (*n* = 21) were exposed to filtered air or DE (100 μg/m^3^) for 2 h, on two separate occasions. Bronchial biopsies were taken 18 h post-exposure and immunohistochemically analysed for pro-apoptotic and anti-apoptotic proteins (Bad, Bak, p85 PARP, Fas, Bcl-2) and a marker of proliferation (Ki67). Positive staining was assessed within the epithelium using computerized image analysis.

**Results:**

No evidence of epithelial apoptosis or proliferation was observed in healthy, allergic or asthmatic airways following DE challenge.

**Conclusion:**

In the present study, we investigated whether DE exposure would affect markers of proliferation and apoptosis in the bronchial epithelium of asthmatics, rhinitics and healthy controls, providing a mechanistic basis for the reported increased airway sensitivity in asthmatics to air pollutants. In this first *in vivo* exposure investigation, we found no evidence of diesel exhaust-induced effects on these processes in the subject groups investigated.

## Background

Outdoor air pollution is of growing concern worldwide, and is linked to an increase in both cardiovascular and respiratory disease [1]. Children, the elderly and subjects with pre-existing respiratory or cardiovascular diseases have been demonstrated to be susceptible groups [2–4]. There is evidence that exacerbations of pre-existing asthma are attributed to exposure to pollutants related to road traffic [5]. Diesel exhaust exposures have also been shown to induce oxidative injury to the airways, leading to inflammation and increased risk of sensitisation [6].

In an attempt to understand the mechanisms responsible for these adverse health effects in humans, controlled exposure studies employing diesel exhaust (DE) have been performed both in healthy individuals and in subjects with established allergic diseases (asthma and rhinitis). These studies have shown that healthy individuals respond acutely to DE challenge with a marked neutrophilic airway inflammation [7–11]. In asthmatics, increased bronchial hyperresponsiveness to methacholine is evident following exposure to high ambient DE concentrations [12]. However, this occurs with no evidence of augmented airway inflammation in terms of diesel exhaust-induced changes in submucosal inflammatory cell number or adhesion molecule expression [7]. The only responses reported in the asthmatic airway following DE have been increased epithelial expression of IL-10 [13] and enhanced sputum IL-6 levels [12]. In another study, asthmatics were exposed to DE in combination with cat allergen, again without any signs of an enhanced airway inflammation [14].

The bronchial epithelium forms a barrier between the external environment and the underlying lung mucosa. Epithelial integrity is usually maintained due to a balance between apoptotic and proliferative processes [15], with the relative activation of the positive and negative regulatory pathways of apoptosis ultimately determining cell fate [15]. The Bcl-2 family are key regulators of the mitochondrial apoptosis pathway and include the pro-apoptosis members Bad and Bak and the anti-apoptosis member Bcl-2 [15–17]. Another pathway of importance is the TNFα receptor pathway, which includes the receptor Fas. Binding to these receptors leads to activation of initiator caspases. These two pathways are also termed the intrinsic and extrinsic pathways and they converge at the effector caspases, including caspase 3, and lead to cleavage of poly (ADP-ribose) polymerase (PARP) into a C terminal 85 kDa peptide fragment [18].

There have been some studies in human subjects, or samples derived from humans, investigating apoptosis in asthma and following exposure to DE. Evidence supports increased apoptosis in asthma, in that the damaged bronchial epithelium found in asthmatics has been related to the degree of bronchial hyperresponsiveness [19, 20]. In a study by Bucchieri employing bronchial biopsies from asthmatics, increased epithelial apoptosis was demonstrated in terms of a greater expression of the caspase cleavage product p85 PARP, compared to healthy controls [21]. Furthermore, using primary epithelial cell cultures, the asthmatic epithelium was shown to be more susceptible to oxidant-induced apoptosis compared with normal controls [21]. *In vitro* studies using epithelial cell lines (A549) have demonstrated that exposure to particulate matter (PM) leads to increased apoptosis [22–24]. Apoptosis is further enhanced in cystic fibrosis epithelial cell lines compared to controls [25]. The effects of PM on the apoptosis pathway *in vivo* and on subjects with pre-existing lung disease are unknown. The lack of an increased airway inflammation following exposure to DE has led us to hypothesize that other mechanisms, apart from airway inflammatory responses, such as apoptosis, may account for the clinical outcomes observed in asthmatics following exposure to traffic-derived pollution.

In the present study, we hypothesised that increased apoptosis, or impaired proliferative responses could potentially enhance the penetration of bronchoconstrictive stimuli to the airway wall, potentially explaining the increased sensitivity of asthmatics to symptoms during periods of high pollution. The specific aim in this study was to explore whether DE-exposure would affect pro-apoptotic and anti-apoptotic proteins and markers of proliferative responses in the bronchial airways. This study included both mild and moderate asthmatics along with healthy controls, as previously described [7]. In addition, due to the observed differential responses to DE in asthmatics and healthy control subjects, atopic non-asthmatics (subjects with allergic rhinitis) were also included. We and others have shown evidence of lower airway inflammation of atopic non-asthmatics that is intermediary between that observed in the bronchial mucosa of asthmatics versus healthy subjects [26–28]. Furthermore, in a controlled DE exposure study of allergic rhinitics, we found that the DE-induced airway inflammatory response was similar to that observed in asthmatic airways [29].

## Methods

This current study was performed as a follow-up investigation of a larger study addressing DE-induced responses in the airways of healthy, rhinitic and asthmatic subjects [7, 29], using archived biopsies. Subjects included healthy controls (non-asthmatic, non-atopic) (*n* = 21), allergic rhinitics without asthma (atopic controls) (*n* = 13), asthmatics on short acting β-_2_ agonists on demand (*n* = 16) and asthmatics on inhaled corticosteroids (200–1200 μg daily) (*n* = 16). Baseline demographics of each group are summarized in Table 1. All participants gave their informed consent and the local ethics committee of Umeå University approved the study, which was performed in accordance with the declaration of Helsinki.

**Table 1:** Baseline demographics of the subjects in each study group

	Healthy controls	Allergic rhinitis	Allergic asthma β2-agonists on demand	Allergic asthma ICS-treatment
Number in group	21	13	16	16
Mean age years (range)	24 (21–29)	25 (22–34)	24 (18–32)	24 (19–41)
Gender (male:female)	12:9	6:7	8:8	8:8
FEV_1_ (% predicted)	98 ± 15	97 ± 15	99 ± 15	93 ± 8
PC_20_ (mg/mL)^a^	Not assessed	>8.0	2.7 ± 1.8	>8.0
Skin prick test to pollens	Negative	Positive	Positive	Positive
SM eosinophils (cells/mlx10^4^)^a^	0.0 (0.0-0.0)	0.4 (0.0-1.2)	0.1 (0.0-1.2)	0.0 (0.0-0.3)
SM mast cells (cells/mlx10^4^)	19 (11–27)	16 (14–28)	27 (18–31)	16 (12–22)
SM neutrophils (cells/mlx10^4^)^a^	61 (30–78)	57 (26–102)	47 (19–97)	98 (75–124)

SM = Submucosa. ^a^SM-eosinophils and SM neutrophils were significantly different between the four groups, Kruskal-Wallis Test 0.017 and 0.033 respectively. Data previously published [7, 29]

Subjects were exposed to filtered air or diesel exhaust (100 μg/m^3^) for 2 h, on two separate occasions, at least 3 weeks apart, in a randomised order, as previously described [7, 29]. During the exposures, subjects alternated exercise on a bicycle ergometer (minute ventilation = 20 L/min/m^2^ body surface area) with rest at 15-min intervals. This exposure setup was used to model a moderate level of outdoor activity in a high-polluted environment. The diesel exhaust was generated from an idling 1991 Volvo diesel engine (Volvo TD45, 4.5 l, four cylinders, 680 rpm). The air within the exposure chamber was monitored continuously and the steady state concentration of PM_10_, gases and semi-volatiles during the diesel exposures were 100 ± 12 μg/m^3^ for PM_10_, 9.1 ± 2.6 parts per million (ppm) for carbon monoxide, 1.2 ± 0.1 ppm for nitrogen monoxide, 0.39 ± 0.05 ppm for nitrogen dioxide, 1.7 ± 0.4 ppm for oxides of nitrogen and 1.1 ± 0.8 ppm for total gaseous hydrocarbons (C3H8-equivalents), expressed as mean ± SD. The PM mass was dominated by fine and ultrafine particles (<1 μm), and the mass median particle diameter was 0.18 μm. Further details of the methods used are described in detail in previous papers [7, 29].

Bronchoscopy with collection of bronchial biopsies was performed at the Department of Medicine, Division of Respiratory Medicine and Allergy, University Hospital, Umeå, Sweden, 18 h after exposure. The biopsies were processed into glycol methacrylate (GMA) resin for immunohistochemistry using standardised protocols [30]. Immunohistochemical staining was performed using the streptavidin biotin-peroxidase technique and monoclonal antibodies directed against the pro-apoptotic markers, Bad (Serotec, Kidlington, UK) and Bak (Serotec); the anti-apoptotic marker Bcl-2 (Dako, Ely, UK); the death receptor Fas (CD95) (TCS Biosciences ltd, Botolph Claydon, UK); the caspase cleavage product p85 PARP (Promega, Southampton, UK); and the cell proliferation marker Ki67 (Dako). Staining was visualised with diaminobenzidine (DAB) and sections counterstained with haematoxylin.

Positive staining was analysed in lengths of intact well orientated epithelium with the assistance of computerised image analysis (KS400 software with a Zeiss Axioskop 2 microscope and Axiocam, Zeiss, Bicester, UK). For Ki67 the percentage positive nuclear area was determined and for all other markers the percentage positive of the total epithelium was assessed, based on the red/green/blue (RGB) colour composition of the DAB staining [31]. In brief, following standardisation of the computerised image analysis system for light level and white balance digitised images of the epithelium were captured. Threshold RGB settings for the DAB staining were applied and adjusted to select all the positive staining within the section. For epithelial markers, the area of the epithelium was then delineated interactively and the percentage of positive staining within the epithelium calculated. For Ki67, DAB positive thresholding was measured against RGB thresholding for blue nuclear counterstaining.

Data were not normally distributed and are therefore presented as medians with interquartile ranges (IQR). All paired air versus diesel exhaust comparisons were performed using the Wilcoxon signed rank test. The Kruskall Wallis ANOVA test was initially used to test for differences in the response (change) to diesel exhaust versus air between the four subject groups and differences at baseline. If a significant difference was observed between the four groups, the Mann Whitney U test was used for further analyses. All statistical analyses were performed using SPSS, version 21.0 (SPSS, Cary, North Carolina, USA). A *p*-value of <0.05 was considered statistically significant.

## Results

Following DE exposure a marked neutrophilic airway inflammation was detected in healthy subjects, a response that was absent in atopic individuals, as previously reported [7, 29].

**Fig. 1:**
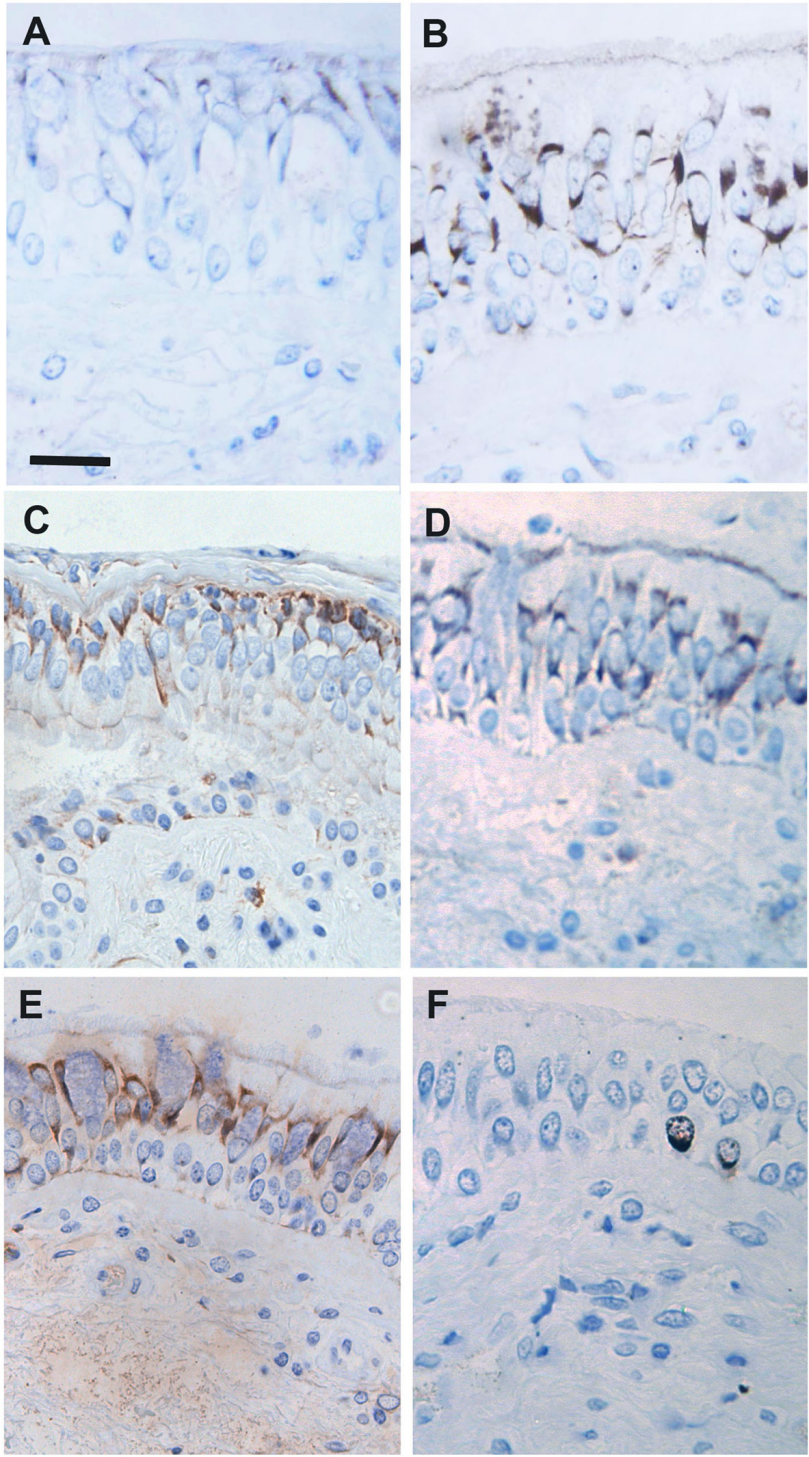
Immunohistochemical staining. Photographs showing the epithelial expression of the pro-apoptotic markers Bad (**a**) and Bak (**b**), anti-apoptotic marker Bcl-2 (**c**), the death receptor Fas (**d**), the caspase cleavage product p85 PARP (**e**) and the proliferation marker Ki67 (**f**). Positive staining is brown, Scale bar is 20 μm

**Fig. 2:**
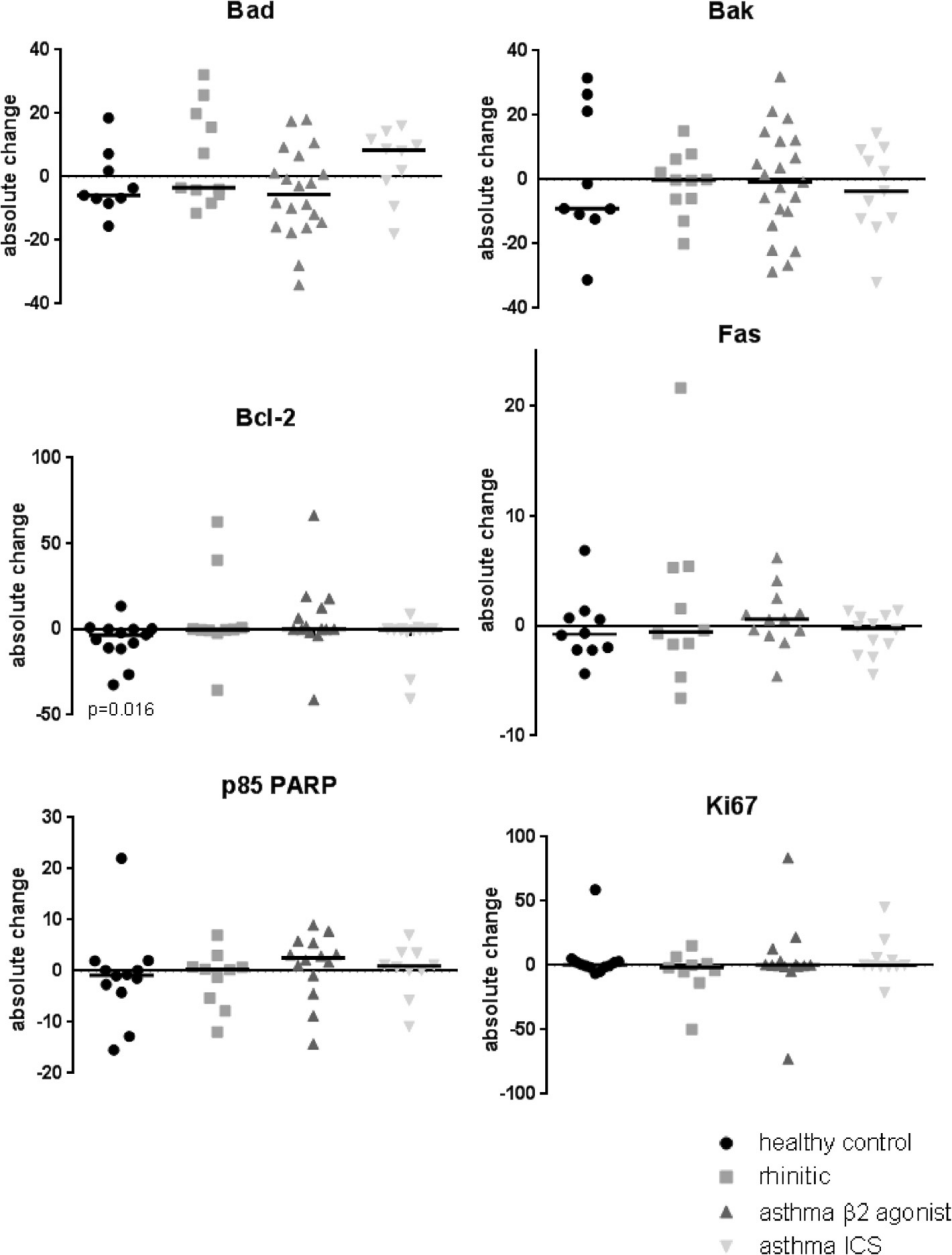
Changes in expression of pro- and anti-apoptotic markers following exposure to diesel exhaust and air. Graphs showing the percentage change in epithelial expression of the pro-apoptotic markers Bad and Bak; the anti-apoptotic marker Bcl-2; the death receptor Fas; the caspase cleavage product p85 PARP; and the proliferation marker Ki67 in the bronchial epithelium of healthy controls (●), allergic rhinitics () and asthmatics on β2 agonists () and on inhaled corticosteroids () 18 h after a 2 h exposure to diesel exhaust (100 μg/m^3^) compared to filtered air. Median values (−) for each group and significant *p* values are shown

Representative images of the immunohistochemical staining are shown in Fig. 1. There were no differences in baseline (post air exposure) in the levels of any of the markers of apoptosis or proliferation examined between the four subject groups (Table 2). In the asthmatic and rhinitic subjects, there was also no significant change in expression (response) following exposure to DE compared to air in any of these markers. However, in the healthy control subjects the expression of Bcl-2 was significantly lowered (*p* = 0.02) after DE compared to filtered air: 4.5 % (IQR 0–11.44) after air *versus* 0.06 % (IQR 0.01–0.62) following DE exposure. However, comparison of the response (change) to diesel versus air exposure across the groups was not significant for Bcl-2 or any of the other markers investigated (Fig. 2).

**Table 2:** Data from immunohistochemistry staining of bronchial biopsies for markers of proliferation and apoptosis

Study group		Healthy controls		Allergic rhinitics		Asthmatics β2		Asthmatics ICS	
Exposure		AIR	DE	AIR	DE	AIR	DE	AIR	DE
Pro-apoptotic markers	Bad	10.8 (5.5 - 20.1)	11.8 (9.1 - 23.3)	29.8 (9.6 - 34.2)	14.7 (4.0 - 27.4)	12.4 (6.0 - 20.7)	21.5 (4.3 - 41.4)	14.4 (6.2 - 22.8)	19.0 (10.7 - 28.8)
	Bak	15.4 (3.8 - 36.4)	17.2 (9.1 - 28.0)	18.9 (8.8 - 31.7)	26.4 (8.3 - 31.9)	14.5 (10.3 - 33.7)	27.0 (13.1 - 38.5)	21.7 (13.3 - 33.2)	23.8 (8.6 - 28.7)
Anti-apoptotic marker	Bcl-2	4.5 (0–11.4)	0.06* (0.01 - 0.6)	0.36 (0.19 - 1.2)	0.07 (0–44.2)	0.34 (0.02 - 5.1)	2.8 (0–17.1)	0.49 (0–3.3)	0.08 (0–5.1)
Death receptor	Fas	2.8 (1.4 - 4.6)	1.4 (0.4 - 5.5)	1.8 (1.0 - 5.9)	1.7 (0.3 - 10.8)	1.9 (0.7 - 3.3)	1.8 (0.4 - 3.0)	1.3 (0.5 - 2.7)	2.0 (0.5 - 1.2)
Caspase cleavage product	P85 PARP	1.7 (0.6 - 6.8)	1.7 (1.0 - 2.8)	3.8 (0.6 - 9.2)	2.6 (0.8 - 6.0)	2.2 (0.4 - 6.9)	4.3 (1.1 - 7.2)	2.1 (0.3- 4.6)	2.6 (0.1 - 4.1)
Proliferation marker	Ki67	1.9 (0.1 - 5.0)	0.7 (0.3 - 3.6)	1.7 (0.4 - 14.6)	11.2 (1.5 - 34.1)	3.1 (0.6 - 10.2)	1.3 (0.2 - 3.4)	1.2 (0.2 - 11.9)	1.7 (0.9 - 8.0)

Data is given as the % positive staining within the epithelium calculated (median percentages and inter quartile ranges)

Within group significant difference between AIR and diesel exhaust (DE) exposure, Wilcoxon Signed Ranks test. * *p* = 0.02

## Discussion

In this study, we investigated the hypothesis that DE would lead to an increase in apoptosis accompanied by impaired repair reflected by decreased proliferation in the bronchial epithelium of asthmatics, providing a mechanistic basis for their reported increased sensitivity to air pollutants. Contrary to our hypothesis, in this first *in vivo* exposure investigation, we found no evidence of the induction of these processes in either mild-moderate asthmatics or allergic rhinitics following DE challenge. Whilst we observed a decrease in Bcl-2 in the healthy subjects following diesel exposure compared to air exposure, no significant changes were found when comparing the Bcl-2 responses across the groups.

Previous DE exposure studies have used different exposure setups. We have reported a marked neutrophilic inflammatory response in the airways of healthy volunteers following exposure to 300 μg/m^3^ DE for one hour, 6 h post-exposure [11]. In a previous study in which healthy and asthmatics were exposed to DE at PM_10_ 108 μg/m^3^ for 2 h, an increase in airway resistance of similar magnitude was observed in both healthy and asthmatics. Healthy subjects also developed airway inflammation 6 h after DE exposure, a response that was absent in asthmatic subjects. However, epithelial staining for the cytokine IL-10 was increased after DE in the asthmatic group, a cytokine with potential anti-inflammatory effects [13]. In the present study, we hypothesised that the inflammatory response would be delayed in the allergic/asthmatics subjects. We therefore exposed them to this lower concentration (100 μg/m^3^) for 2 h and performed the bronchoscopies 18 h post exposure. The dose was chosen to mimic real world exposure more closely. This is also a dose observed at kerbside locations for example in London (http://www.londonair.org.uk/). Again we found a neutrophilic airway inflammation in the healthy subjects but not in subjects with allergic rhinitis or asthma. We therefore hypothesised that other mechanisms such as apoptosis would be affected by the DE exposure.

The failure of this study to confirm the previous *in vitro* studies demonstrating increased apoptosis in A549 cells in response to PM could suggest that these responses do not extrapolate to the *in vivo* setting or may reflect differences in methodologies between *in vitro* and *in vivo* systems [22–25]. The A549 cell line is a transformed adenocarcinoma alveolar type II cell line. These cells are not normal and have a squamous phenotype; therefore they are likely to respond differently to PM than the *in vivo* stratified columnar bronchial epithelium. Also, these investigators used different methodologies to assess apoptosis, measuring different end-points and features of apoptosis, which again may account for the diverse outcomes observed. Their techniques included: TUNEL (terminal deoxynucleotidyl transferase-mediated dUTP nick-end labelling) to assess DNA fragmentation [22, 23]; measurement of DNA nucleosomal fragmentation by ELISA; [23, 25] colourimetric assays for caspase 3 and 9 activation; [25] and Western blot to measure active caspase 3 and fragmentation of cytokeratins. In the immortalised BEAS-2B bronchial epithelial cell line, which resembles the *in vivo* bronchial epithelium more closely, no effect on apoptosis following exposure to DE was observed [32], which concurs with our findings. In their study, Cao et al. measured antibody immunoreactivity to detect Bcl2 and p85 protein by Western blotting, which is similar to the immunohistochemical approach we employed.

There was variability in the expression of the markers explored both at baseline (Table 1) and following DE exposure (Table 2 and Fig. 2). It is well known that asthma has a heterogeneous immunopathology that could to some extent impact on this variability. However, this did not result in any outliers in clinical responses following DE exposure. The participants in the present study were all stable in their disease. If the study had included subjects with uncontrolled asthma, the results may have been different.

Whilst none of the examined markers were altered in the asthmatics or allergic rhinitics after exposure to DE, this study is the first to report an *in vivo* effect of DE exposure on Bcl-2 expression in healthy subjects. However, as this change in expression was not significant in comparison with the other subject groups, this finding should be considered with caution. Bcl-2 is known to be a key apoptosis regulatory protein of the mitochondrial death pathway, and has an anti-apoptotic function that is closely associated with its level of expression [33]. Oxidative stress has been shown to downregulate Bcl-2 expression and thus promote apoptosis [34]. Exposure of A549 cells to parabenzoquinone, a component of DE, leads to decreased expression of Bcl-2 [35]. Also, over-expression of Bcl-2 has been shown to delay apoptosis of macrophages induced by chemical extracts from DE [36] and to promote survival of cultured retinal pigment epithelial cells exposed to oxidative damage [37].

The observed decrease in the anti-apoptotic marker Bcl-2 would suggest an increase in apoptosis following acute exposure to oxidative air pollution, which may be a protective mechanism to remove damaged epithelium. It has also been suggested that decreased apoptosis, if accompanied by increased proliferation, may be a repair response to protect the epithelium [38]. However, in the present study, the decrease in Bcl-2 was not paralleled by an increase in p85 PARP, and so we could therefore not confirm that exposure to DE leads to increased apoptosis, or an increase in Ki67 expression that would suggest a proliferative response.

Whilst the lack of a measurable response to DE within the bronchial epithelium in the asthmatic subjects may reflect a difference between *in vitro* and *in vivo* exposure (as discussed earlier), it is possible this may be due to epithelial shedding of damaged cells. An impaired epithelial barrier due to epithelial shedding [19, 20, 39] is a characteristic feature of asthma, as is increased sensitivity to oxidative damage [21]. In the present study the number of epithelial cells in bronchial lavages was very low, and there were no signs of epithelial shedding in any of the groups. Neither was there any sign of shredding in the biopsy material. Another possible scenario could have been that the exposure of an already fragile sensitive epithelium to DE could already have led to apoptosis and shedding into the bronchial lumen by 18 h. It may therefore be more appropriate to look at an earlier time point after DE exposure. In this study we did not observe a response assessed by changes in markers of apoptosis or proliferation in the bronchial epithelium of the allergic rhinitic subjects when exposed to DE, suggesting their responses are similar to those seen in asthmatics.

Whilst previous studies have reported increased markers of apoptosis and proliferation in asthmatics [40–42], we did not observe any differences in the expression of the pro-apoptotic markers, Bad and Bak; the anti-apoptotic marker Bcl-2; the death receptor Fas; the caspase cleavage product p85 PARP; or the cell proliferation marker Ki67 between the subject groups when exposed to air. Druilhe reports increased expression of Fas in the bronchial epithelium of asthmatics compared to controls, but no difference in Fas L, Bcl-2 or PCNA [40]. p85 PARP was found by Western blot in asthmatic epithelial cells, accompanied by an increase in immunohistochemical staining for Ki67. Such responses were not seen in healthy controls [41]. In another study, Bcl-2 expression was increased in the bronchial epithelium of asthmatic subjects compared to controls, implying an anti-apoptotic effect, but in the absence of PCNA positivity [42]. Following an asthma exacerbation, an increase in Bcl-2 expression in BAL lymphocytes has been shown, but contrary to our study, these asthmatics had a FEV_1_ that ranged between 62 and 83 % predicted, compared with 98 % in our current study. The increase in Bcl-2 could therefore be related to asthma severity [43]. There are also other previous studies employing less severe asthmatics that also failed to demonstrate differences in Bcl-2, Fas or Ki67 compared to healthy controls [44].

## Conclusion

These initial data do not support our original contention that the heightened sensitivity of asthmatics to traffic derived particulate matter might reflect an underlying imbalance between cell clearance and proliferation in response to DE; effectively enhancing the penetration of bronchoconstrictive stimuli to the airway wall. We acknowledge that this interpretation is limited by the single time point examined, and further studies will be required before we can fully exclude the involvement of these pathways in the induction of airway hyper-responsiveness after exposure to particulate matter air pollution.

## Abbreviations

DAB: Diamino benzidene
DE: Diesel exhaust
FEV_1_: The volume exhaled during the first second of a forced expiratory maneuver
GMA: Glycol methacrylate
IQR: Interquartile range
PARP: Poly ADP-ribose polymerase
PC_20_: Histamine concentration causing a 20 % drop in FEV_1_
PCNA: Proliferating cell nuclear antigen
PM: Particulate matter
